# A Comparative Study between Postoperative Analgesia of Fascia Iliaca Compartment Block and Anterior Quadratus Lumborum Block in Proximal Femur Fracture

**DOI:** 10.1155/2022/3465537

**Published:** 2022-05-17

**Authors:** O. S. M. Abd Elmaksoud, S. E. M. Elansary, N. G. Fahmy, R. M. Hussien

**Affiliations:** Intensive Care and Pain Management, Faculty of Medicine, Ain Shams University, Cairo, Egypt

## Abstract

Elderly patients with various comorbidities are more likely to suffer from proximal femur fractures. It is also a painful fracture, and poor pain management can have serious physiological and psychological consequences, such as acute delirium. *Purpose*. The aim of this study is to compare the efficacy of ultrasound-guided transmuscular (anterior) quadratus lumborum block (QLB) versus infrainguinal fascia iliaca compartment block (FICB) in proximal femur fractures for postoperative analgesia. Patient-reported pain on the visual analogue scale (VAS), analgesic demand, and ambulation were the key factors. *Patients and Methods*. This prospective, randomised trial was done after receiving approval from the institute' study ethical committee. In this study, ultrasound-guided infrainguinal fascia iliaca compartment block was compared to ultrasound-guided anterior quadratus lumborum block using 50 ml of bupivacaine 0.25%, with a maximum dose of 2.5 mg/kg at the end of surgery after spinal anaesthesia in 128 patients (64 patients in each group). Nalbuphine was given as rescue analgesia if VAS >3. Our 1^st^ outcome was the first rescue analgesia and total analgesic consumption in the 1^st^ 24 hours; the 2^nd^ outcome was the time patients started to ambulate. *Results*. Postoperative pain perception was substantially greater in the FICB group starting from 30 min (*P* value 0.022) till 24 hours (*P* value <0.001), and they received a considerably larger total narcotic dose (14.1 ± 3.5) than patients in the QLB group (7.9 ± 3.4), *P* value (<0.001^*∗*^). The time required to achieve first rescue analgesia was much less in the FICB group (8.5 ± 2.2) compared to the QLB group (14.1 ± 4.5), *P* value (<0.001^*∗*^), and they took much longer to ambulate (22.3 ± 4.8) when compared to the QLB group (20.1 ± 4.6), *P* value (0.011^*∗*^). Hypotension (1.6%) was detected mainly in the QLB group, whereas poor fascial separation (1.6%) was observed only in the FICB group. There were no significant differences in complications between both the FICB and QLB groups. *Conclusion*. Patients receiving postoperative anterior QL block for proximal femur fracture demonstrated delayed first rescue analgesia and lower total nalbuphine consumption with early ambulation than patients who received FICB.

## 1. Introduction

Pain is the unpleasant sensory and emotional experience that is linked to tissue injury that has occurred or may occur. This concept emphasises that pain is always subjective, with each individual learning about pain through early life experiences involving tissue injury, when pain is reported in terms of severity, location, and sometimes quality [1].

Fracture neck of femur frequently affects elderly population who usually suffer from multiple comorbidities. There are considerations about offering good analgesia while controlling for the dose of opioid used [2]. The search for better analgesia using less invasive techniques has increased the popularity of interfascial plane blocks [3, 4].

Fascia iliaca compartment block (FICB) is a pain-relieving method that includes infusing local anaesthetics under the sheath of the iliacus muscle [5]. FICB can be performed either directed by ultrasound or with a deficiency of the opposition (LOR) method [6].

The quadratus lumborum block (QLB) is a regional anaesthesia technique described in 2007 aiming at providing adequate analgesia for abdominal surgeries. Later, it was found that this block also provides analgesia to the hip. Moreover, various reports have demonstrated its efficacy in the setting of femoral neck femur fracture [7, 8].

Both techniques were used successfully in providing adequate analgesia in patients undergoing hip surgery. However, to our knowledge, postoperative analgesia provided by infrainguinal fascia iliaca compartment block and transmuscular (anterior) quadratus lumborum block in patients undergoing proximal femur fracture fixation following spinal anaesthesia has not been compared before regarding their analgesic efficiency.

Our hypnosis is that since ant QLB has more spread and affection to the lumbar plexus than infrainguinal FCIB, blockage of lat cut the nerve of the thigh and iliohypogastric (site of surgical incision) is more granted with ant QLB, which guarantee a longer period of postoperative analgesia.

This study aims to analyse the effectiveness of ultrasound-guided transmuscular (anterior) quadratus lumborum block (QLB) versus infrainguinal fascia iliaca compartment block (FICB) for postoperative analgesia in proximal femur fractures. Patient-reported pain on the visual analogue scale (VAS), analgesic demand, and ambulation were the key factors.

## 2. Materials and Methods

This prospective, randomised comparative trial was conducted after obtaining the approval of the institution's research ethics committee, including patients of both sexes. American Society of Anesthesiologists (ASA) physical status II–IV, aged ≥50 years old, and scheduled for fracture femur surgeries under spinal anaesthesia.

**Figure 1:**
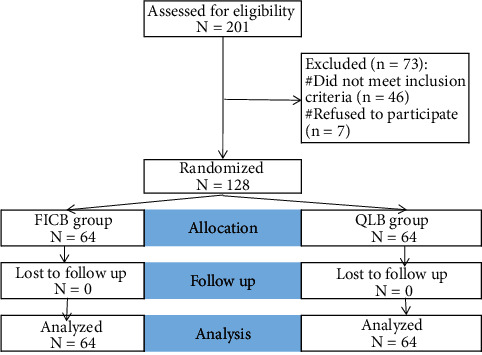
Flowchart of the studied cases.

Patients with severe spine abnormalities, bleeding disorders and coagulopathy, preexisting myopathy or neuropathy, and significant cognitive dysfunction, those with infection at the injection site, known to be allergic to local anaesthetic, patients with many fractures who took long-acting opioids prior to surgery, and those who had a failed spinal anaesthesia were excluded from the study, as shown in Figure 1.

Using G power software for sample size calculation and according to Kinjo S et al. [9], using the first rescue analgesia and total analgesic consumption in the 1^st^ 24 hours as a primary outcome, it is estimated that the sample size of 128 patients (64/group) can achieve 80% power to detect a statistical significance between two groups regarding quantitative outcome measures (VAS score and analgesic dose) for medium effect size corresponding to a Cohen *d* coefficient of 0.5 using the two-sided Student's *t*-test with alpha error 0.05.

Patients fulfilling the inclusion criteria were randomised into two equal groups by a computer-generated random numbers table, each consisting of 64 patients, namely, group I and group Q. Patients of both groups received spinal anaesthesia where patients lied in the lateral decubitus; the back was disinfected by povidone-iodine and covered with drapes; after identification of L4-L5 or L3-L4 level, 1 ml of 2% lidocaine was injected subcutaneously at the point of needle insertion, a spinal needle G25 was then advanced, and heavy Marcaine 20 mg was injected intrathecally on the appearance of CSF. Patients were given general anaesthesia if spinal anaesthesia failed, and they were excluded from the study.

Group I: patients received ultrasound-guided infrainguinal fascia iliaca compartment block; where at the end of the operation, patients lay supine, the skin was disinfected, and a high-frequency linear probe was put transversely on the inguinal crease, after identification of the femoral artery, the fascia iliaca, and iliopsoas muscle. The probe was moved laterally till identifying the sartorius muscle. A 22-gauge needle was inserted in-plane through fascia iliaca; then, 50 ml of 0.25% bupivacaine (not exceeding a toxic dose of bupivacaine 2.5 mg/kg) was injected within the fascial plane between the fascia iliaca and the iliopsoas muscle.

Group Q: patients received ultrasound-guided anterior quadratus lumborum block; where at the end of surgery, patients were put in the lateral decubitus, the skin was disinfected, and a curved low-frequency ultrasound probe was put in a vertical position just above the iliac crest; on the identification of quadratus lumborum and psoas major (PM) muscles, a 22-gauge needle advanced in-plane from the posterior end of the curved probe, traversing the QL till its tip was seen between the PM and the QL muscles; then, 50 ml of 0.25% bupivacaine (not exceeding a toxic dose of bupivacaine 2.5 mg/kg) was injected into the fascial plane.

At the end of the procedure, patients were transported to the postoperative intermediate care unit, where patients were monitored and observed for any complications arising from the procedure, such as hematomas, or from the drugs injected, such as hypotension, bradycardia, a drop in peripheral oxygen saturation, nausea, vomiting, or any other adverse effect that would be dealt with appropriately. Postoperative pain was assessed by the visual analogue scale (VAS).

In case of hypotension (a reduction in blood pressure of 20% or more from baseline), intravenous ephedrine (10–30 mg diluted in 10 ml normal saline 0.9%) was titrated to the desired blood pressure. 0.5 mg of atropine was given in the instance of bradycardia (HR < 60) that was coupled with hypotension or any signs of poor perfusion. Supplemental oxygen was supplied in the event of a drop in peripheral SpO_2_ to keep it above 94%. In case of postoperative nausea and vomiting (PONV), 4 mg ondansetron diluted in 10 mL 0.9% normal saline was given intravenously over 10 minutes.

The block was considered a failed block if the visual analogue scale (VAS) was more than three after the analgesic effect of spinal anaesthesia faded (Bromage score 1 of the healthy limb), i.e., if the patient can move the healthy limb and there is pain at the site of surgery.

A VAS of more than three was managed by giving nalbuphine 5 mg intravenously in addition to intravenous paracetamol (10–15 mg/kg) IV every 8 hours.

The study's primary goal was to evaluate the time it took for the two groups to request analgesics for the first time, and the secondary objective was to compare total opioid consumption, ambulation time, and nerve block complications over 48 hours.

### 2.1. Statistical Methods

IBM SPSS (Statistical Package for Social Sciences) software version 22.0, IBM Corp., Chicago, USA, was used to code, tabulate, and statistically analyse the obtained data. After testing for normality using the Shapiro–Wilk test, quantitative normally distributed data were reported as the lowest and maximum of the range and the mean SD (standard deviation) and then compared using an independent *t*-test if normally distributed. For variables with modest expected numbers, qualitative data were compared using the chi-square test and Fisher's exact test expressed in numbers and percentages. The log-rank test was used to compare the rates of rescue analgesia and ambulation. If the *P* value is less than 0.050, the result is significant; otherwise, it is nonsignificant.

## 3. Results

This study reveals no statistically significant variations in baseline characteristics such as age, sex, ASA, and operation type between the FICB and QLB groups, as given in Table 1. It also showed that postoperative pain perception was substantially more remarkable in the FICB group than in the QLB group commencing at minute 30. At hours 8 and 24, the maximum gabs of pain scores were seen between the study groups, as given in Table 2.

The FICB group received a considerably larger total narcotic dose than the QLB group. Time required to achieve first rescue analgesia was much less in the FICB group compared to the QLB group. Patients in the FICB group took much longer to ambulate than those in the QLB group, as given in Table 3.

Hypotension was detected mainly in the QLB group, whereas poor fascial separation was observed only in the FICB group. There were no significant differences in complications between both the FICB and QLB groups, as given in Table 4.

**Figure 2:**
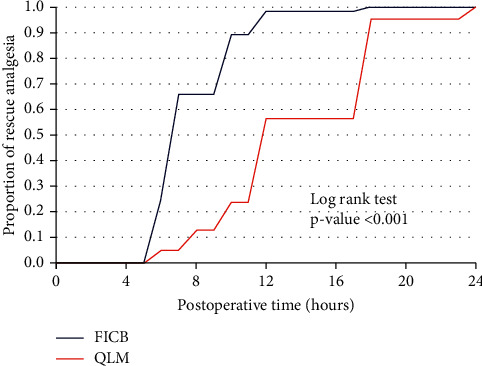
Kaplan–Meier curve for first rescue analgesia.

**Figure 3:**
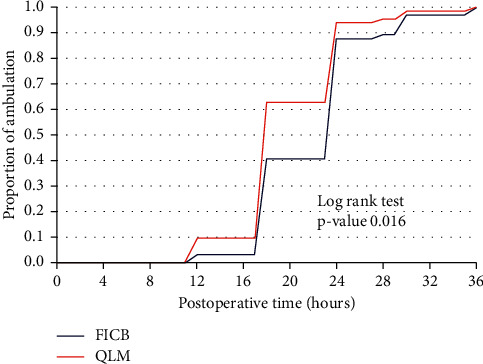
Kaplan–Meier curve for mobilization.

The rate of rescue analgesia was significantly greater in the FICB group compared to the QLB group as shown in Figure 2, and the rate of ambulation was significantly slower in the FICB group than in the QLB group as shown in Figure 3.

Table 1 provides that there are no statistically significant differences between FICB and QLB groups regarding baseline characteristics: age, sex, ASA, and operation type.

Table 2 and Figure 1 show that postoperative pain perception beginning from hour 6 onwards was significantly higher in the FICB group than in the QLM group. The maximum gabs of pain score between the studied groups were at hours 8 and 24.

Total narcotic dose was significantly higher in the FICB group than in the QLB group. Time to first rescue analgesia was significantly shorter in the FICB group than in the QLB group. Onset of ambulation was significantly longer in the FICB group than in the QLB group.

Figure 1 shows that the rate of rescue analgesia was significantly higher in the FICB group than in the QLB group. Figure 2 shows that the rate of ambulation was significantly slower in the FICB group than in the QLB group.

Hypotension was recorded only in the QLB group. Bad separation of fascia was recorded only in the FICB group. There were no significant differences in FICB and QLB groups regarding complications.

## 4. Discussion

In our study, we compared infrainguinal FICB and anterior QLB in terms of postoperative analgesia in elderly patients with fractured neck femur; both blocks were performed at the end of the surgery, by the same anesthesiologist and by a known surgical team. We found that patients who received QLB had lower VAS pain scores in the first postoperative hours and a lower analgesic demand during the first 24 hours, as well as earlier ambulation when compared to infrainguinal FICB. We attributed the better analgesia provided by QLB to the better spread of local anaesthetic, especially the transmuscular approach of QLB that affects lumbar plexus better than FICB.

The quest for optimal analgesia with less invasive techniques while decreased opioid consumption has increased the popularity of neuraxial techniques and ultrasound- (US-) guided interfacial plane blocks in elderly patients with fractured necks of femur [3, 4]. Fascia iliaca compartment block (FICB) is an anterior regional anaesthetic block of the lumbar plexus in which local anaesthetics are injected posterior to the fascia iliaca, causing the local anaesthetic to diffuse through its layers and subsequently to the femoral, genitofemoral, lateral femoral cutaneous, and obturator nerves. Monzon et al. [10] provided analgesia for areas innervated by lumbar plexus branches, including the skin, muscles, periosteum, hip, thigh, and knee joints [11]. It has also been used in the prehospital setting for pain management, showing a high success rate and fewer complications [12].

On the other hand, the quadratus lumborum block (QLB) is a posterior abdominal wall block that enables the local anaesthetic to diffuse posterior to the quadratus lumborum muscle and spread past the middle layer of the thoracolumbar fascia into the lumbar interfacial triangle [13, 14], and it is useful in treating high-risk elderly patients suffering from proximal femoral fractures [15, 16]. Current literature on the QL block describes four different approaches. Ueshima et al. [13] reported that for (lateral, posterior, anterior, and intramuscular) QL block, in the lateral QLB, local anaesthetic is injected lateral to the QL muscle with the diffusion of local anaesthetic between QL and transversalis fascia, posterior QLB, the injection is posterior to the QL muscle. The transmuscular QLB (anterior QLB) involves injecting the local anaesthetic at the anterior aspect of the QL muscle which can expand cranially beneath the lateral arcuate ligament towards the endothoracic fascia reaching the lower thoracic paravertebral space posterior to the endothoracic fascia. The anterior QL block may provide analgesia from T10 to L4, which affords analgesia for both the trunk and the lower extremities, unlike the lateral and posterior QLB, which provides analgesia from T7 to L1 that may be useful in the treatment of perioperative pain after abdominal surgery. So, QL3 was performed in this study [13].

In the transmuscular (anterior) quadratus lumborum block, the local anaesthetic is administered between the psoas major (PM) and the QL muscles [17] causing analgesia from T10 to L4 [18,19] due to blockage of the lumbar plexus, so it was chosen in our study despite being technically difficult when compared to other approaches.

Many studies have been carried out to find a way to decrease opioid consumptions. Yun et al. and Madabushi et al. [20, 21] reported that both blocks showed superior analgesia to intravenous opioids. These studies demonstrated that FICB delivers superior analgesia to intravenous fentanyl. Yun et al., Madabushi et al. [20, 21], and others reported that FICB analgesia was superior to that induced by intravenous morphine [22]. Moreover, the employment of QLB in hip surgery revealed a reduction in hospital stay and fentanyl consumption intraoperatively [23].

Neuraxial analgesia via epidural or spinal techniques was frequently used, and while this approach ensured adequate analgesia, it can result in delayed ambulation, which is considered a disadvantage, as early mobilization is strongly recommended for the pathway of enhanced recovery following surgery [24]. On comparing epidural analgesia to continuous FICB, in patients undergoing fracture neck femur fixation, patient-controlled epidural analgesia produced better analgesia than patient-controlled fascia iliaca block [25]. However, according to Murdoch et al., continuous epidural infusion of local anaesthetics is frequently associated with hypotension [26].

On comparing the quadratus lumborum block to neuraxial blocks, it was found that QLB provided more prolonged analgesia than spinal anaesthesia alone in two separate studies [17, 27]. Likewise, these findings indicated that adopting QLB as the default technique may markedly reduce opioid consumption and complications following hip surgery and that the main benefit of QLB over other regional anaesthesia techniques is the greater spread of local anaesthetic agents in anterior QLB beyond the transversus abdominis plane to the thoracic paravertebral space, which results in extensive analgesia and prolonged action of local anaesthetic agents [18]. Furthermore, QLB may have a role in multimodal analgesia for patients undergoing hip surgery due to its analgesic efficacy while preserving muscle strength, which makes early functional rehabilitation unlikely to be impaired. Bugada et al. [28] corroborate La Colla et al.' case reports where QL block was given in two cases of fracture neck femur and redo hip arthroplasty and demonstrating that it offered significant analgesia without causing weakness in hip flexor or quadriceps muscle, making it superior to epidural analgesia. [29].

Fowler et al. stated that various techniques of peripheral nerve blocks, as the femoral nerve block (FNB) and the lumbar plexus block (LPB), given in total hip arthroplasty procedures, were expected to cause less serious complications than epidural anaesthesia [30]. However, Njathi et al. stated that peripheral nerve blocks might be associated with an increased risk of serious adverse effects such as nerve injury and hematoma due to the advancement of the needle tip near the nerves [31]. FCIB blocks femoral, obturator, genitofemoral, and lateral femoral cutaneous nerves better than 3 in 1 block [32] and was confirmed by Capdevila et al., who stated that FCIB was faster and more concordant at blocking femoral and lateral femoral nerves in lower limb surgeries [33].

Additionally, studies that compared anterior QLB to lumbar plexus block for total hip surgeries found that there was no difference in IV morphine equivalent consumption or pain scores [34, 35]. Moreover, anterior QLB provides analgesia from T10 to L4 nerve roots with good cephalic spread better than LPB in a study on cadavers using dye injections [36]. Ryan et al. demonstrated that QLB was superior to femoral nerve and fascia iliaca blocks in decreasing opioid consumption perioperatively, providing better pain scores, and decreasing discharge time after hip arthroscopy [37]. However, Brixel et al. pointed out that the sensory blockade associated with QLB was frequently patchy and does not correspond to a classic dermatomal distribution [38]. While, Kinjo et al. reported that QLBs did not provide adequate analgesia for hip arthroscopy for femoroacetabular impingement when compared to other regional blocks. However, they related the small sample size they included in their experimental group as a limitation, and they suggested that a slight change in block location may cause variable anaesthetic diffusion [9]. So, in this study, a larger sample size was chosen to address this issue.

In comparison to FICB, QLB results in lower VAS pain scores in the first postoperative hours and a lower analgesic demand during the first 24 hours, as well as earlier ambulation. This may be explained by the cephalic spread to the paravertebral space, which resembles LBP without hemodynamic and muscle weakness. Additionally, QLB provides blockage of the genitofemoral and iliohypogastric nerves, which arise so early in the lumbar plexus and are unaffected by FICB.

## 5. Conclusion

Patients receiving postoperative anterior QL block for proximal femur fractures demonstrated delayed first rescue analgesia and consumed less total nalbuphine with early ambulation than those who received FICB.

### 5.1. Limitation

In our hospital, the more common and much easier practice was inferior FCIB that may spare the lateral cutaneous nerve block despite our usage of large volume.

## Figures and Tables

**Table 1:** Baseline characteristics of the studied groups.

Variables		FICB (*N* = 64)	QLB (*N* = 64)	*P* value
Age (years), mean ± SD		69.5 ± 10.6	68.1 ± 9.5	^^^0.426

Sex (*n*, %)	Male	34 (53.1%)	38 (59.4%)	^#^0.476
	Female	30 (46.9%)	26 (40.6%)	

ASA (*n*, %)	I	22 (34.4%)	25 (39.1%)	^#^0.510
	II	28 (43.8%)	30 (46.9%)	
	III	14 (21.9%)	9 (14.1%)	

Operation type (*n*, %)	Intertrochanteric	36 (56.3%)	44 (68.8%)	^#^0.317
	Neck	18 (28.1%)	14 (21.9%)	
	Subtrochanteric	10 (15.6%)	6 (9.4%)	

^^^Independent *t*-test. ^#^Chi-square test.

**Table 2:** Postoperative pain perception (VAS-10) among the studied groups.

Time	FICB (*N* = 64)	QLB (*N* = 64)	^*P* value	Effect size	
				Mean ± SE	95% CI
Minute 0	0.0 ± 0.0	0.0 ± 0.0	0.999	0.0 ± 0.0	0.0–0.0
Minute 15	0.0 ± 0.2	0.0 ± 0.0	0.165	0.0 ± 0.0	0.0–0.1
Minute 30	0.1 ± 0.2	0.0 ± 0.0	0.083	0.1 ± 0.0	0.0–0.1
Minute 45	0.1 ± 0.2	0.0 ± 0.0	0.083	0.1 ± 0.0	0.0–0.2
Hour 1	0.2 ± 0.4	0.1 ± 0.2	0.055	0.1 ± 0.1	0.1–0.2
Hour 2	0.4 ± 0.6	0.3 ± 0.5	0.055	0.2 ± 0.1	0.0–0.4
Hour 4	1.1 ± 0.8	0.9 ± 0.7	0.056	0.3 ± 0.1	0.0–0.5
Hour 6	3.3 ± 1.3	1.8 ± 1.0	<0.001^*∗*^	1.5 ± 0.2	1.1–1.9
Hour 8	3.8 ± 1.4	2.4 ± 1.0	<0.001^*∗*^	1.4 ± 0.2	1.0–1.9
Hour 10	3.5 ± 1.3	3.0 ± 1.0	0.012^*∗*^	0.5 ± 0.2	0.1–0.9
Hour 12	4.4 ± 1.2	3.7 ± 1.5	0.003^*∗*^	0.7 ± 0.2	0.2–1.2
Hour 18	5.3 ± 1.3	4.3 ± 1.4	<0.001^*∗*^	1.0 ± 0.2	0.5–1.5
Hour 24	5.7 ± 1.3	3.8 ± 1.3	<0.001^*∗*^	1.9 ± 0.2	1.4–2.3

Data presented as mean ± SD unless mentioned otherwise. ^Independent *t*-test. ^*∗*^Significant. Effect size: value of FICB relative to QLB. SE, standard error; CI, confidence interval.

**Table 3:** Total narcotic dose and time to first rescue analgesia and ambulation among the studied groups.

Measures	FICB (*N* = 64)	QLB (*N* = 64)	^^^ *P* value	Effect size	
				Mean ± SE	95% CI
Total narcotic dose (mg)	14.1 ± 3.5	7.9 ± 3.4	<0.001^*∗*^	6.2 ± 0.6	5.0–7.4
Time to first rescue analgesia (hours)	8.5 ± 2.2	14.1 ± 4.5	<0.001^*∗*^	−5.6 ± 0.6	−6.8–4.4
Onset of ambulation (hours)	22.3 ± 4.8	20.1 ± 4.6	0.011^*∗*^	2.2 ± 0.8	0.5–3.8

^Independent *t*-test. ^*∗*^Significant. Effect size: value of FICB relative to QLB. SE, standard error; CI, confidence interval.

**Table 4:** Complications among the studied groups.

Complications	FICB (*N* = 64)	QLB (*N* = 64)	^§^ *P* value	Effect size Relative risk 95% CI
Subcutaneous collection	1 (1.6%)	1 (1.6%)	0.999	1.00 (0.06–15.64)
Bad separation of fascia	1 (1.6%)	0 (0.0%)	0.999	Not applicable
Hypotension	0 (0.0%)	1 (1.6%)	0.999	Not applicable

^§^Fisher's exact test. Effect size: value of FICB relative to QLB. CI, confidence interval.

## Data Availability

The datasets used to support this study are available from the corresponding author upon request.
